# Diverse Roles for Hyaluronan and Hyaluronan Receptors in the Developing and Adult Nervous System

**DOI:** 10.3390/ijms21175988

**Published:** 2020-08-20

**Authors:** Alec Peters, Larry S. Sherman

**Affiliations:** 1 Division of Neuroscience, Oregon National Primate Research Center, 505 NW 185th Avenue, Beaverton, OR 97006, USA; peteral@ohsu.edu; 2 Department of Cell, Developmental and Cancer Biology, Oregon Health & Science University, 3181 SW Sam Jackson Park Rd, Portland, OR 97239, USA

**Keywords:** hyaluronic acid, hyaluronan, nervous system, neurodevelopment, neurogenesis, extracellular matrix

## Abstract

Hyaluronic acid (HA) plays a vital role in the extracellular matrix of neural tissues. Originally thought to hydrate tissues and provide mechanical support, it is now clear that HA is also a complex signaling molecule that can regulate cell processes in the developing and adult nervous systems. Signaling properties are determined by molecular weight, bound proteins, and signal transduction through specific receptors. HA signaling regulates processes such as proliferation, differentiation, migration, and process extension in a variety of cell types including neural stem cells, neurons, astrocytes, microglia, and oligodendrocyte progenitors. The synthesis and catabolism of HA and the expression of HA receptors are altered in disease and influence neuroinflammation and disease pathogenesis. This review discusses the roles of HA, its synthesis and breakdown, as well as receptor expression in neurodevelopment, nervous system function and disease.

## 1. Introduction

Hyaluronic acid (HA), or hyaluronan, is a linear unsulfated glycosaminoglycan composed of repeating units of D-glucuronic acid and N-acetyl-D-glucosamine that can be thousands of residues in length and megadaltons in size [1,2]. HA is a major component of the extracellular matrix (ECM) in many tissues including the central nervous system (CNS) [3,4,5]. HA has a diverse set of mechanical functions in tissues, such as providing elasticity in skin and acting as a lubricant between articulating bones in joints [5]. However, this relatively simple glycosaminoglycan also acts as a complex extracellular signal that can regulate a diverse set of cell processes based on its size, associated proteins, and interactions with receptors.

Hyaluronan has been found to regulate cellular activities in development, homeostasis, and disease states in a variety of tissues including the nervous system, where it has been implicated in regulating neuronal and glial cell differentiation, the activity of neurons, and nervous system repair in neurodegenerative diseases and following CNS injuries. These functions depend on tightly regulated changes in HA synthesis and catabolism. In this review, we will discuss recent findings supporting distinct roles for HA in each of these processes, and we examine evidence that HA acts as a dynamic signaling molecule that can influence diverse activities in the developing and mature mammalian brain.

## 2. Hyaluronic Acid Acts as Both a Structural Element of the Extracellular Matrix and a Signaling Molecule Regulating Multiple Cellular Processes

### 2.1. High Molecular Weight HA Has Functions in the Nervous System That Are Distinct from HA Fragments

Native extracellular HA can be large (megadaltons in size) and is often termed high molecular weight (HMW) HA. Through hyaluronidase activity or reactions with reactive oxygen species, HMW HA can be broken down into fragments of varying sizes [5,6]. HMW HA and fragments of different sizes can influence a variety of biological processes through interactions with receptors such as CD44, the receptor for hyaluronan-mediated motility (RHAMM, otherwise known as hyaluronan-mediated motility receptor or HMMR), and lymphatic vessel endothelial hyaluronan receptor 1 (LYVE1). HA can also activate toll-like receptors (TLRs) 2 and 4, although it is currently unclear if this occurs through direct HA binding [2,7].

Different sizes of HA can have distinct biological activities. HMW HA is known to reduce inflammation in a variety of tissues, while fragments of different sizes either promote or inhibit inflammatory processes [6,8]. HMW HA also reduces proliferation of multiple neural cell types [9,10,11,12,13], and both HMW HA and HA fragments can regulate cell differentiation in the CNS [12,13,14,15]. Additionally, HA signaling can change based on its association with other ECM components as well as covalent modifications. HA-associated proteins and protein complexes, such as the proteoglycans aggrecan, brevican, and neurocan, and link proteins, such as Bral1, Bral2, and HAPLN1, associate with HA in the nervous system ECM [16,17,18,19]. These large HA-protein complexes help to regulate nervous system functions including cell adhesion, migration, and neurite outgrowth. HA is also found in the proteoglycans that form the specialized ECM around neurons called perineuronal nets (PNN) [20,21,22,23,24,25,26,27,28,29,30]. Covalent modifications to HA, such as the addition of the inter-α-trypsin inhibitor (IαI) heavy chain (HC) catalyzed by tumor necrosis factor stimulated gene 6 (TSG-6) also changes the signaling properties of HA and can modulate neuroinflammation, HA crosslinking, and interactions between HA and receptors [31,32,33,34,35,36].

### 2.2. HA Is Required for Nervous System Development

HA is found in the CNS at the earliest stages of development, as the neural plate folds into the neural tube. The amounts of HA peak in the embryonic nervous system then decline in adulthood [37,38], although the absolute amounts of HA in any area of the CNS or PNS have yet to be quantified at any stage of life. The treatment of embryos with a hyaluronidase to digest HMW HA led to delays in neurulation, linking HA to some of the earliest morphogenic processes during neurodevelopment [39].

After neurulation, the lowest concentration of HA is found in the ventricular zone where neural stem cells (NSCs) are undergoing self-renewal [13]. HA receptors including CD44 and RHAMM are also expressed in the ventricular zone, and RNA analyses of the developing CNS indicate that cell–ECM interactions through these and other ECM receptors may be crucial for the proliferative capacity of NSCs [40,41,42]. A higher concentration of HA is found in the intermediate zone where stem cells differentiate into neuroblasts [13], though it is unclear if the higher HA concentrations are inducing differentiation or acting in a different capacity such as providing a permissive environment for the migration of cells that have already begun to differentiate.

During later stages of neurodevelopment, HA is required for proper neocortical folding [43]. HA has also been shown to be involved in neurite extension and proper axon routing, such as that of retinal neuron axons in the optic pathway [44,45,46], as well as cell migration in the developing cerebellum [47]. More studies are required to fully understand how changes in HA and HA-mediated signaling contribute to CNS morphogenesis.

In addition to early CNS development, HA is required for early events in peripheral nervous system (PNS) development. HA is found around the neural tube, notochord, and neural crest after neurulation [19,48,49]. Neural crest cells express HA synthases (HAS) 1 and 2, and this expression is downregulated during migration [50]. Neural crest cells also express CD44 and RHAMM [40,51]. HAS expression, notably HAS2, as well as HA is also found along neural crest migratory paths [19,52]. Knockdown of CD44 delays neural crest migration [51], and silencing of HAS2 during neural crest migration induces migratory defects [53]. All together, these findings indicate that HA has crucial roles in the initiation of neural crest cell emigration from the dorsal neural tube and in migration as these cells differentiate into neurons, Schwann cells and other peripheral nerve cell populations.

### 2.3. HA Is Required for Mature Nervous System Function

HA is a nearly ubiquitous component of the extracellular matrix in the adult CNS, found in both gray and white matter. HA is found surrounding myelinated axons in white matter, while in a gray matter, it is found in a more diffused distribution as part of the ECM and as a component of PNN surrounding neurons [17,54,55]. HA concentrations vary by brain region, as shown by studies observing distinct HA staining patterns in the somatosensory and piriform cortices [56], as well as the cerebellum [57]. HA has diverse structural and signaling functions in the nervous system, such as forming a scaffold for the development of PNNs in gray matter [58] while having a critical structural role in myelinated axons and axon fiber tracts in white matter [54,55,59,60], as well as in myelinated axons of the PNS [59].

Adult neurogenic niches, including the subgranular zone (SGZ) of the hippocampal dentate gyrus and the subventricular zone (SVZ), harbor NSCs that undergo self-renewal as well as asymmetric division to form new neurons and glial cells. HA is a major extracellular component in both of these niches [12,61]. HA has been linked to cell quiescence in many in vitro and in vivo studies in both NSCs and glia [9,10,11,12,13,62]. HA signaling through receptors such as CD44 and TLR2 help to regulate stem cell proliferation and differentiation [12,63], and loss of CD44 leads to hippocampal dysfunction and spatial learning and memory deficits [64]. This suggests an essential role for HA signaling in adult neurogenesis, as well as behaviors that rely on incorporating new neurons into neural circuits throughout life. In vitro studies indicate that embryonic neural stem cells have hyaluronidase activity [65], suggesting that neural stem cells could possibly regulate neurogenesis through the modulation of HA concentration and fragment size in neurogenic niches. It is possible then that learning and memory lead to activity-dependent changes in HA synthesis and hyaluronidase activity that regulate the generation of new neurons and neuronal maturation.

HA and HA signaling has also been shown to be correlated with cell migration in the adult nervous system. HA is elevated in the rostral migratory stream, a specialized migratory route along which neuronal precursors that originated in the SVZ migrate to reach the olfactory bulb. Migrating immature neurons in the rostral migratory stream express RHAMM, indicating that HA-RHAMM interactions may be required for the proper migration of these cells [61].

In vitro studies with 3D HA hydrogels have found that the stiffness of the ECM can influence properties of neural stem and progenitor cells. For example, by modulating HA concentration to generate hydrogels that mimic elasticities of the developing and adult brain, it was found that an elasticity near that of the developing embryonic brain permitted neuronal progenitor migration and branching, while higher elasticities mimicking the adult brain did not [65]. Furthermore, 3D hydrogels with higher HA concentration had decreased stem cell proliferation and differentiation [62,65]. The stiffness of the ECM could also promote proliferation, migration, and branching of one cell lineage over another [66] and can even direct stem cell differentiation into different lineages [67]. It is possible that gradients of ECM can guide cell migration from areas of lower to higher stiffness [68]. Although how HA influences ECM stiffness in CNS tissues is not fully elucidated, HA-binding proteins such as TSG-6 might play a role in modulating HA crosslinking and changes to the stiffness of the ECM. The size of the component HA can also directly influence ECM stiffness either directly or through modulating TSG-induced crosslinking of HA strands [31,69]. These findings suggest that changes in extracellular HA concentration during development can lead to cell responses by changing the mechanical properties of the ECM as well as through interactions with HA receptors. Modifying the mechanical properties of the ECM by altering HA size and concentration could also help regulate neurogenesis in adult neurogenic niches.

In addition to regulating the expansion and differentiation of neural stem cell populations, HA can also regulate receptor function in both CNS and PNS neurons. Direct interactions between HA and the transient receptor potential cation channel subfamily V member 1 (TrpV1; also known as the capsaicin receptor) expressed by dorsal root ganglion neurons decreased receptor activation and sensitization, leading to reduced responses to capsaicin and heat [70]. Loss of HA through hyaluronidase administration to the footpad of rats increased pain sensitivity in a CD44 dependent manner [71], suggesting that HA can also influence neuronal activity at sensory neuron endings. HA has also been found to modulate the activities of CNS neurons. For example, hyaluronidase administration reduced L-type voltage gated calcium channel activity in hippocampal neurons [72], as well as increasing AMPA receptor trafficking [73]. These findings raise the possibility that PNNs or similar structures may be dynamically regulated to alter neuronal activity.

The role of HA in PNNs has been reviewed previously [74], and so the role of PNNs, their development, and the function of HA in these structures will only be briefly reviewed here. HA is a critical component of most PNNs along with chondroitin sulfate proteoglycans (CSPGs) and link proteins. HA forms a scaffold in which these other ECM components can be organized onto to form fully functional PNNs [75]. While originally thought to be synthesized by glia or a combination of glia and developing neurons [76], other studies reported that developing neurons alone express HAS enzymes and synthesize PNNs [58,77]. However, glial cells and neurons may synthesize different components to form fully functional PNNs in different regions of the brain [78].

Cultured developing neurons slowly generate PNNs over the course of days [58,77], mimicking the slow development and maturation of these structures in the developing CNS [17,54,79,80,81]. In the developing CNS, PNNs form in a region-specific manner that correlates with functional maturation [79]. PNNs mostly surround parvalbumin expressing inhibitory neurons [58,82], and it is thought that synthesis and maturation of PNNs leads to decreased synaptic plasticity [81], possibly due to restricting lateral diffusion of receptors such as AMPA receptors to specific areas on neuron cell bodies including postsynaptic densities [73]. This idea is reinforced through studies that show that the removal of PNNs through hyaluronidase treatment increased excitatory neuronal activity as well as changing receptor subunit expression to those observed during critical periods [81,83]. Loss of PNNs through hyaluronidase treatment also increased the seizure-like activity of neurons in vitro [84] and increased the rates of seizures in animal models [85]. Interestingly, the prefrontal cortex in schizophrenics displayed decreased PNNs [86]. These observations suggest that developmental deficits in HA synthesis leading to decreased PNN formation could lead to CNS disorders such as epilepsy and schizophrenia.

In addition to its effects on progenitor cells and neurons, HA can also influence the behaviors of glial cells. Astrocytes express HAS enzymes and synthesize HA [61,76,87]. Similar to NSCs, extracellular HMW HA seems to be a quiescence signal for astrocytes both in vitro [10,65] and in vivo [9], as well as for O2A progenitors that give rise to type II astrocytes [11]. HA has been shown to regulate the migration and morphology of astrocytes though interactions with CD44 and RHAMM [88,89,90]. The proper trafficking and function of glutamate transporters in astrocytes also rely on HA [91]. During late embryonic and postnatal development, HA production by astrocytes could help to form the extracellular matrix as well as decrease proliferation of neighboring astrocytes and, in neurogenic niches, neural stem cells.

The few studies to investigate HA changes with aging have reported increases in HA content in older nervous systems [12,92,93,94,95]. The increased HA observed in aged brains is synthesized by different cell types, including astrocytes and cells of the microvasculature [93,94]. Both the cause of this excess HA synthesis and the effects of the increased HA remain unclear. Given that increased HA levels generally lead to cell quiescence, reduced cell proliferation is a likely outcome. In neurogenic niches such as the SGZ, increased HA content could be one of the causes of significantly decreased neurogenesis observed in older brains [96].

### 2.4. HA in Injury and Disease

Increases in both HMW HA and HA fragments have been found in a variety of CNS insults including trauma, dementia, ischemia, and inflammatory demyelinating diseases such as multiple sclerosis (MS) [97,98,99]. During trauma and disease, HMW HA can regulate astrocyte proliferation and glial scar formation as well as microglial activation [9,10,100,101]. Additionally, HMW HA impacts vascular endothelial cell barrier function [102] and contributes to lymphocyte extravasation and the onset of experimental autoimmune encephalomyelitis (EAE)—a model of MS [103]. HA fragments can influence angiogenesis [104,105], neural stem cell proliferation [63], and differentiation of neural progenitor cells such as oligodendrocyte progenitor cells (OPCs) under pathological conditions [15,106,107].

HA binding proteins such as CSPGs are found in glial scars and help form a barrier that reduces the spread of inflammatory cells but also contribute to the impairment of axonal regeneration. Other binding proteins such as TSG-6 are upregulated in CNS insults such as injury and Alzheimer’s disease [34,35,108]. TSG-6 is involved in the induction of reactive astrocytes in a model of traumatic brain injury, but it also has been shown to inhibit microglial activation in vitro [35,36]. TSG-6 could exert its effects through the covalent modification of HA and the formation of HA-HC complexes, the crosslinking of HA, and regulating HA interactions with receptors such as CD44, all of which could change the signaling properties of HA in nervous system insults.

Interestingly, the effects of TSG-6 are dependent on the sizes of HA and the presence of IαI. For example, TSG-6 catalyzed bonding of HC onto HA molecules is reversible in HMW HA, but HC is irreversibly bound to small HA fragments (4–12 oligosaccharide polymers) [33]. Additionally, IαI inhibits HA crosslinking by TSG-6 [109]. This suggests that the effects of TSG-6 on HA can be modulated in injury and disease through the breakdown of HMW HA and expression of IαI to generate different cell responses.

While changes in HA size, structure, and binding proteins may be beneficial in the case of trauma to reduce the amount of neuronal damage due to inflammation, in other cases such as chronic diseases, changes in HA amount and size may be detrimental to regeneration. For instance, in white matter diseases such as MS and vanishing white matter disease, accumulation of HMW HA and HA fragments in plaques have been found to inhibit the maturation of OPCs and functional remyelination [15,106,107,110].

## 3. HA Synthesis Is Tightly Regulated in the Nervous System

### 3.1. Hyaluronic Acid Synthase Proteins and Their Distribution in the CNS

HA in mammalian cells is synthesized by three HA synthases (HAS1–3)—transmembrane enzymes localized to the plasma membrane of cells that extrude HA into the extracellular space during synthesis [111]. HAS activity increases pericellular HA levels, and each of the three HAS enzymes have different kinetics and synthesize different size ranges of HA [111]. The expression patterns of each HAS isoform are not identical throughout development, in the adult and in old age. This suggests that HA of different size ranges synthesized by each of the different HAS isoforms play distinct roles in the development and functioning of the nervous system through differential signaling of their respective products. Supporting this are studies showing that silencing of different HAS genes leads to opposite effects on cell motility in vitro [112].

There are some differences in HAS expression patterns in different species. In developing *Xenopus* embryos, HAS1 is expressed throughout the ectoderm, excluding the neural plate, while HAS2 is expressed in mesoderm, and HAS3 is restricted to a small number of structures such as the developing otic vesicles [50]. In bovine embryos, while HAS2 and HAS3 are expressed throughout embryonic development, HAS1 is only expressed in early (2- and 4-cell) stages [113]. In mice, HAS1 is widely expressed in early development until about embryonic day 8.5, when its levels are markedly decreased [52]. HAS2 expression continues and is eventually restricted to specific structures, including the developing heart and in craniofacial development, while HAS3 expression appears at later times in structures such as developing teeth and hair follicles [52]. Human embryonic stem cell lines express HAS2 as they are induced to form blastocysts [114], and this influences their differentiation into extraembryonic cell types [14,114]. HAS proteins, therefore, play critical roles in early development that may be distinct in different species.

### 3.2. HAS Regulation in the Nervous System

HAS enzymes synthesize pericellular HA matrices in vitro that differ depending on which enzyme is expressed [111,115]. Cultured primary cortical neurons express all three HAS isoforms, with HAS3 having the highest expression [77]. HAS enzymes play an important role in PNN development by synthesizing the HA that serves as the backbone for PNN formation surrounding neurons. HAS3 co-expressed with other components of PNNs in human embryonic kidney cells led to the synthesis of a compact extracellular matrix that was similar in structure to neuronal PNNs [75]. It is possible that HAS enzymes are an integral part of the PNN and not simply synthesizing the HA backbone of PNNs, as there is evidence that HA can be tethered to the cell membrane by HAS enzymes and not just receptors such as CD44 [116]. HAS1 and HAS2 are differentially expressed in neural crest cells, and the silencing of either enzyme delays neural crest migration [51].

In the aging nervous system, accumulation of HA is correlated with increases in different HAS protein expression based on location and cell type. HA accumulation in aged gray matter around astrocytes is linked to increased HAS1 expression [93], while HA accumulation around cortical microvasculature is associated with increased expression of HAS2 [94]. These studies show that in developing, adult, and aging nervous systems, the three HAS enzymes have different spatiotemporal expression and generally nonoverlapping functions.

### 3.3. Pharmacologic and Genetic Inhibition of HA Synthesis Impacts Nervous System Development and Function

While a HAS2 global knockout is embryonic lethal, loss of either HAS1 or HAS3 leads to normal development with no obvious morphological changes [117], further supporting the idea that HAS enzymes have distinct functions in neurodevelopment. However, there are some contexts in which the other HAS isoforms can compensate for the lack of one. For example, only the loss of all three isoforms abolishes glutamate transporter localization to the tips of astrocyte cell processes [91]. HAS3 knockout mice and mice lacking HAS2 in the nervous system through a nestin-driven conditional knockout lack HA in different brain regions. While HAS3 knockout mice have less HA in the hippocampus, HAS2 conditional knockout animals have reduced HA levels in the cortex and white matter, including the corpus callosum [117]. HAS2 also plays a role in the development of myelin sheaths, as knockout mice display a greater number of myelin sheath abnormalities resulting in less compact myelin lamellae and decreased axon diameter [60].

Interestingly, inhibition of HAS activity through the administration of 4-methyl-umbelliferone, which inhibits hyaluronan synthesis by depletion of cellular UDP-glucuronic acid and, possibly, by the downregulation of HAS2 and HAS3 expression, delayed the onset and severity of EAE [118]. These findings suggest that increased hyaluronan synthesis can potentiate autoimmune neuroinflammatory events.

## 4. Regulation of HA Catabolism in the Nervous System

### 4.1. Multiple HA Catabolizing Proteins Are Expressed in the Nervous System

Hyaluronidases and HA catabolizing proteins are dynamically regulated in the nervous system, and it is increasingly clear that fragments produced by hyaluronidase-driven catabolism of HMW HA have distinct signaling roles in the CNS. HA is catabolized by multiple hyaluronidases (i.e., HYAL1–3 and HYAL5/SPAM1/PH20), the products of which can vary in size [119]. HYAL1, 2, 3, and PH20 have sequence and structural homology and catabolize HMW HA using the same basic reaction mechanism, but each one has different subcellular locations and optimal conditions for activity [119]. Another related enzyme, HYAL4, does not have hyaluronidase activity and hydrolyzes chondroitin sulfate [120].

HYAL1 is located in lysosomes, while HYAL2 is GPI anchored to the plasma membrane [121,122]. It is thought that native high molecular weight HA present in the extracellular matrix is partially degraded first outside the cell, and smaller HA sizes are endocytosed and further degraded by HYAL1 in lysosomes. Early studies show that HA turnover and hyaluronidase activity is highest during development [38]; however, it is not completely clear which hyaluronidases play a role in the developing and adult nervous systems.

Two more recently discovered proteins display hyaluronidase activity but are structurally distinct from the other hyaluronidases. These two proteins are CEMIP (cell migration inducing and hyaluronan binding protein, also known as HYBID or KIAA1199) and TMEM2. CEMIP expression has been shown to lead to the degradation of extracellular HA in live cells [123]; however, its expression in the developing nervous system has yet to be fully elucidated. TMEM2 has also been shown to be involved in the digestion of extracellular HA [124], but its role as a hyaluronidase is currently debated, as another study has shown that the silencing of TMEM2 increases the degradation of HA [125]. These two proteins will need further study to identify their functions in the developing and adult nervous systems.

### 4.2. Hyaluronidases Have Diverse Functions in the Nervous System

There is a great deal that is still unknown about the role of hyaluronidases in the development of the nervous system, their functions in the adult nervous system, and how they change with aging. Hyaluronidase activity can influence cells through at least two mechanisms: the reduction in extracellular HMW HA and the production of HA fragments with signaling activity.

HYAL2 expression leads to the degradation of extracellular HA in a CD44 dependent manner [126], and HA binding to CD44 has been shown to recruit HYAL2 and a sodium/hydrogen exchanger NHE1 into a macromolecular complex that causes local acidification and HYAL2-dependent extracellular HA degradation [127]. HYAL2 function has also been associated with cell motility [112]. It is possible that migrating cells use a CD44/NHE1/HYAL2 complex for the localized degradation of extracellular HA in the direction of movement to allow cells to move through an environment that otherwise would not permit migration. On the other hand, other studies have shown that HYAL2 silencing does not abrogate the degradation of extracellular HA [123], indicating that at least under some conditions, HYAL2 is not necessary to degrade HA in the ECM. Furthermore, studies in early development have indicated a role for HYAL2 in blastocyst formation [113], but observations that HYAL2 null animals survive to a later stage in development indicate that HYAL2 is not completely necessary for early embryogenesis.

Mice that are lacking either HYAL1 or HYAL3 show no accumulation of HA in the nervous system, indicating that the roles of these hyaluronidases may not be as significant in the CNS and PNS as in other tissues [128,129]. HYAL2 null mice have severely decreased lifespans due to cardiac defects caused by an accumulation of HA [130]. These mice do exhibit HA accumulation in the CNS; however, the only CNS cells that were shown to express HYAL2 were endothelial cells and cells of the choroid plexus [131].

In contrast to studies of HYAL knockout animals, CEMIP knockout mice show an accumulation of HA in the hippocampus as well as hippocampal dysfunction resulting in spatial learning and memory defects [132,133]. These findings indicate that CEMIP is involved in the catabolism of extracellular HA in the CNS and suggest that HA plays an important role in hippocampal function. However, CEMIP null animals live to adulthood with no other obvious abnormalities, again suggesting that it is not the only hyaluronidase active in neurodevelopment.

### 4.3. Hyaluronidases in CNS Disease

Hyaluronidase expression and activity are increased following a number of insults to the CNS including ischemia [97,99]. The activities of different hyaluronidases can produce HA fragments of different sizes and signaling activities that affect nervous system repair.

OPCs express multiple hyaluronidases including HYAL1, HYAL2, and PH20 [107], although the expression of PH20 in OPCs and the CNS is disputed [134]. HA fragments generated by increased HA depolymerizing activity in injured CNS tissues can block the differentiation of OPCs into myelinating oligodendrocytes to inhibit functional remyelination in inflammatory demyelinating lesions [15,106,107,110]. Blocking hyaluronidase activity using a small molecule inhibitor promoted OPC maturation and functional remyelination [135], though the identity of the hyaluronidase responsible for the catabolism of HA in the ECM and production of these inhibitory fragments is currently unknown. Other than PH20, TMEM2 has been shown to digest extracellular HA [124]; however, its function as a hyaluronidase is currently debated. CEMIP is another candidate under investigation given that the small molecule inhibitor that promotes functional remyelination also blocks HA degradation by CEMIP [135]. More studies will need to be completed to determine the hyaluronidase(s) involved in generating inhibitory HA fragments in CNS disease.

## 5. Multiple HA Receptors Respond to HA in the Developing, Mature, and Diseased Nervous System

### 5.1. CD44, RHAMM, TLR2, and TLR4 Are Expressed throughout the CNS and PNS

Receptors involved in HA signaling are highly regulated during neurodevelopment, aging, and disease. Among these receptors, CD44 is the most studied. CD44 is one of the main HA receptors in tissues [136]. It is a single-pass transmembrane glycoprotein that transmits changes in extracellular HA to the cytoplasm to allow cell responses to alterations in content and abundance of HA [2,136]. CD44 binding to HA in cells is variable and can be regulated based on its expression, alternative splicing, and post-translational modifications. While many alternative splice variants exist with different affinities for HA, one of the most prominent in the nervous system is the standard form CD44s, encoded by exons 1–5 and 16–20 [3,137]. Other splice variants have been reported in certain types of neurons [137]. The functions of CD44 variants in the nervous system, both developing and adult, have yet to be fully elucidated.

CD44s binding to HA is partly dependent on N-glycosylation and sialic acid modifications to the extracellular domain [138,139]. Loss of both N-glycosylation and bound sialic acid moieties increases the binding of HA to CD44, presumably though the selection of a protein folding conformation that has a greater HA affinity [138,139]. Additionally, membrane clustering of CD44 is in part determined by the size of interacting HA molecules, which can regulate downstream signaling and cell responses [140]. HA signaling through CD44 plays multiple roles in the developing and adult nervous system, influencing the cell proliferation, differentiation, migration, and morphology of many different cell types in diverse contexts.

RHAMM (or HMMR) also has multiple functions in both the developing and adult CNS. Originally found to function in cell migration, as the name suggests, more recent studies have ascribed a greater variety of functions for RHAMM. Interestingly, RHAMM is found both on the cell surface and intracellularly [114,141,142], indicating that the cellular location of RHAMM may determine its function. Surface expression of RHAMM could be involved in the regulation of cell migration and process extension through interaction with extracellular HA, while intracellular RHAMM could be involved in microtubule dynamics such as chromosomal segregation and proper mitosis, as well as maintenance of cell polarity. The cellular location and subsequent function of RHAMM may be determined by expression of different isoforms via alternative splicing [142].

While TLRs have been extensively studied as pattern receptors involved in innate immune responses to stimuli such as lipopolysaccharide, double stranded RNA, and other damage and pathogen associated molecular patterns, TLR2 and TLR4 also function in HA signaling involving HMW HA as well as lower molecular weight HA fragments [143]. Although it is unclear if HA binds directly to TLRs, TLR2 and TLR4 have been shown to be involved in various aspects of nervous system development, including cell proliferation and differentiation. HA activation of TLRs may, therefore, be a critical part of the signaling involved in nervous system morphogenesis and function.

### 5.2. HA Receptors in Neurodevelopment and the Adult Nervous System

CD44 is expressed by numerous nervous system cell types [12,41,44,46,103,144,145,146,147], and a variety of functions have been attributed to CD44-HA interactions in each of these cells. CD44 is expressed on neural progenitor cells in the developing CNS and is restricted to subsets of cells as development proceeds [41,144], including adult NSCs [12]. In adult NSCs, CD44 expression is associated with both self-renewal and differentiation into neurons. NSCs lacking CD44 have increased proliferation rates, as well as increased rates of differentiation into immature neurons that display maturation deficits [12], suggesting an essential role of HA signaling though CD44 in regulating adult neurogenesis. CD44- and HA-dependent regulation of adult neurogenesis may explain why CD44 null animals experience learning and memory defects [64]. These findings are consistent with studies showing that CD44 has functions in other embryonic and adult stem/progenitor cell populations [148,149].

In developing embryos, CD44 knockdown was found to delay migration of neural crest cells [51], indicating a role of CD44 signaling in PNS development. Additionally, CD44 was found to be involved in the recognition of axon routing cues. Expression of CD44 was found at the midline of the optic chiasm [44], and the inhibition of CD44 through neutralizing antibodies blocked the crossing of growing retinal neuron axons through the optic chiasm during the development of the optic pathway [46]. In other populations of neurons, CD44 has been shown to play a role in the establishment of dendrite morphology [145], as well as the formation of dendritic spines and functional synapses [146]. Furthermore, antagonism of CD44 reduced long-term potentiation in hippocampal neurons [146], and CD44-HA interactions contribute to pain sensitivity [70], as well as anxiety-like behavior [150].

CD44-HA interactions contribute to lamellipodia outgrowth and increased cell migration [112,150,151,152,153], indicating multiple functions of HA signaling in different cell types and contexts though cytoskeletal rearrangements. Through CD44, HA was found to promote migration in both astrocytes [88] and OPCs [154], as well as contributing to the establishment and maintenance of astrocyte morphology [89]. The ECM could regulate cell migration through the direct binding of HA to CD44, or through changes in extracellular matrix stiffness brought about by increasing local concentrations of HA [155].

RHAMM is expressed during embryonic development in a variety of species [40,114,156]. In developing blastocysts, RHAMM expression has been found to be expressed throughout the ectoderm, becoming restricted to the neural plate and eventually migratory and post migratory neural crest cells and the ventricular zone of the neural tube after neurulation [40]. This suggests a role for RHAMM in regulating both stem cell proliferation and migration. In human embryonic stem cells, RHAMM was shown to function in mitosis as well as maintenance of pluripotency [114]. These effects on stem cell proliferation and pluripotency were also observed in mouse embryonic stem cells, in which RHAMM silencing reduced cell proliferation and increased differentiation through upregulation of the MEK/ERK pathway [141].

RHAMM plays an important role in nervous system development. It is required for proper radial intercalation of neuroepithelial cells and the formation of the neural tube [157]. RHAMM-HA interactions also play an important role in neocortical folding [43]. In the adult CNS, RHAMM has been found to be expressed by neural progenitor cells in HA-enriched neurogenic niches such as the SVZ, as well as migrating neural progenitors in the rostral migratory stream [61]. RHAMM is also expressed by a subset of neurons in the hippocampus [158].

Interactions between HA and RHAMM have been found to regulate process extension and cell migration in both neurons and glial cells. Neurons express RHAMM, and blocking interactions between HA and RHAMM inhibit neuronal migration and neurite extension [159]. There is also evidence that both astrocytes and microglia express RHAMM, and that HA-RHAMM interactions regulate migration in both glial cell types [90].

TLR2 and TLR4 both function in NSC proliferation and neurogenesis. TLR2 expression has been observed on cortical neuron progenitors in the SVZ, and in these cells, stimulation of TLR2 with an agonist decreased cell proliferation in vitro, while in developing brains, TLR2 activation increased ventricular size and decreased the size of proliferative zones [63]. TLR4 expression was observed on neural progenitors in the retina, and the activation of TLR4 decreased cell proliferation and induced the differentiation of progenitors into neurons [160]. Human NSCs express both TLR2 and TLR4, which decreased upon differentiation [161]. TLR4 inhibition decreased human NSC proliferation, while its activation increased differentiation rates [161].

TLR2 and TLR4 are expressed in adult neurogenic niches, such as the hippocampus and SVZ, where TLR2 modulates adult NSC differentiation into different lineages (i.e., neurons or astrocytes), and TLR4 regulates stem cell proliferation [162]. These observations of TLR2 and TLR4 expression by both embryonic and neural stem cells, as well as their involvement with NSC proliferation and differentiation, suggest that both TLRs are involved in the modulation of stem cell proliferation and stem/progenitor cell differentiation throughout life, at least in part through interactions with HMW HA and HA fragments.

OPCs express multiple HA receptors including CD44, TLR2, and TLR4 [15,41,163]. Originally it was found that HMW HA blocked OPC differentiation into oligodendrocytes through interactions with TLR2 [15]. Later studies have shown that HMW HA may regulate OPC differentiation in a TLR2- and CD44-dependent manner while HA fragments of specific size block OPC maturation through TLR4 [15,106,107]. This suggests a mechanism where HMW HA interaction with TLR2 and CD44 potentiate hyaluronidase activity, and the HA fragments produced from this activity go on to inhibit OPC differentiation. HA fragments interacting with OPCs suppress AKT activation through TLR4 and its adaptor TRIF, leading to the dissociation of transcription factors such as Olig2 from myelin gene promoters and their transcriptional repression to ultimately inhibit OPC maturation to myelinating oligodendrocytes [106].

### 5.3. HA Receptors in Nervous System Disease

Receptors for HA regulate multiple cell processes in a variety of nervous system diseases. CD44 expression is elevated in multiple types of CNS insults including ischemia [99], traumatic injury [164,165], Alzheimer’s disease (including multiple splice variants of CD44) [166], Parkinson’s disease [167], and white matter diseases such as MS and animal models of MS [97,98,168]. This increase in expression is primarily observed in astrocytes and microglia [110,147,168]. While CD44 plays a role in inflammation and immune cell infiltration into the CNS during neuroinflammatory events such as EAE, there are conflicting results showing that CD44 attenuation enhances [169] and inhibits [103,170,171] EAE onset and progression. CD44 also regulates OPC migration to sites of injury and OPC differentiation arrest in demyelinated lesions [106,154], indicating a multifaceted role of CD44 in CNS inflammation and disease. RHAMM was also found to be upregulated after ischemic events [61,99], although the consequences of this increased expression are not completely clear.

As mentioned above, CD44, TLR2, and TLR4 are implicated in the inhibition of OPC maturation in demyelinating lesions through interactions with HMW and HA fragments of certain sizes (175 to 300 kDa) [15,106]. TLR4 has also been shown to play an important role in nervous system inflammation after insults such as trauma and ischemia through observations that pharmacological inhibition of TLR4 improves outcomes after these events [163,172,173]. Interestingly, HA tetrasaccharides can improve outcomes after spinal cord injury in a CD44 and TLR4 dependent manner [174], and also protect hippocampal neurons during ischemia through the inhibition of TLR2 signaling [175].

## 6. Conclusions

**Figure 1:**
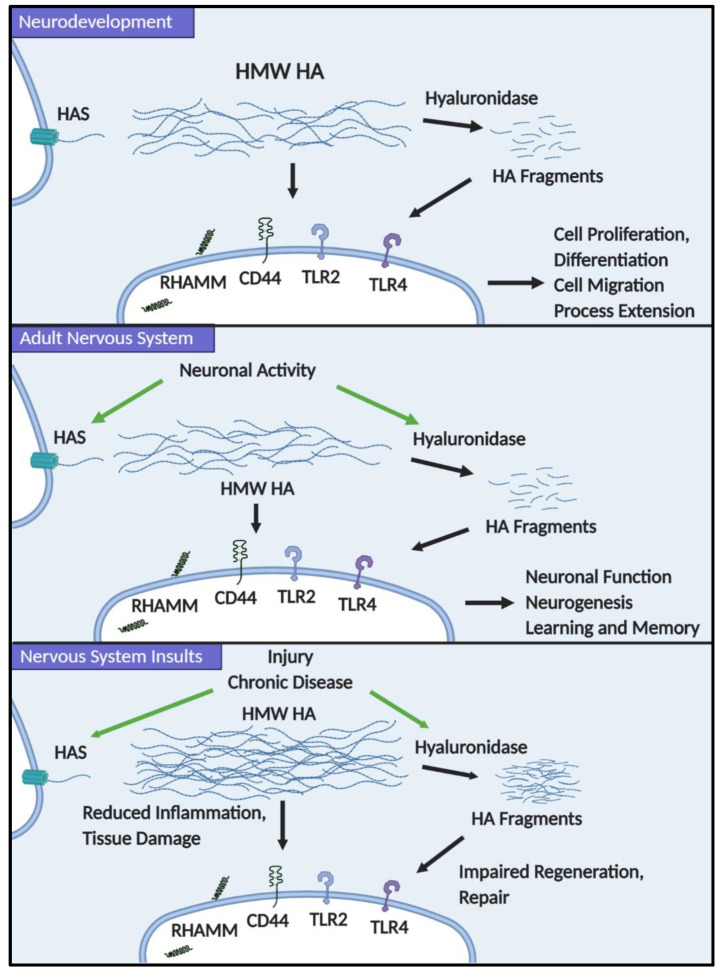
Hyaluronic acid (HA) has diverse functions in neurodevelopment, the adult nervous system, and in nervous system disease. During neurodevelopment, changes to HA in the extracellular matrix (ECM) through the modulation of HA synthases (HAS) and hyaluronidase expression and activity help regulate neural stem and progenitor cell proliferation and differentiation, as well as migration and process extension of different neural cell lineages. In the adult nervous system, neuronal activity can alter HAS and hyaluronidase expression to alter neuronal function and adult neurogenesis. During insults to the nervous system, increased HAS expression and activity leading to high molecular weight (HMW) HA accumulation can modulate neuroinflammatory events and provide protection from tissue damage; however, the buildup of HA fragments from hyaluronidase activity can inhibit the repair capacity of progenitor cells.

We have outlined the many known roles that HA plays in multiple cell types in the developing and adult nervous system (Figure 1). Although much work remains to be conducted to fully understand the different functions of HA in the CNS and PNS, we propose that HA acts both by signaling through HA receptors and through its physical properties to influence the structural features of tissues to regulate neural stem/progenitor cell, neuronal, and glial cell behaviors. In particular, the balance of HA synthesis and catabolism must be regulated during nervous system development, providing cues that influence neural stem/progenitor cell expansion and differentiation, and then, cell migration and process extension of differentiating neurons and glia. In the mature nervous system, we propose that HAS and hyaluronidase gene expression is influenced by neuronal activity, leading to the dynamic regulation of HA synthesis and degradation to influence neuron function but also processes such as neurogenesis during learning and memory. Finally, this dynamic regulation of HA is altered following nervous insults, initially leading to increased HA synthesis that may temper inflammatory responses that cause nervous system damage, but later resulting in the accumulation of HA fragments that feed back on progenitor cell populations to limit their repair capacity (e.g., limiting OPC maturation by blocking myelin gene expression). This framework provides numerous opportunities to explore signaling cascades that are influenced by changes in the HA-based ECM and possible targets to promote nervous system repair following injury and in neurodegenerative diseases.

## Abbreviations

HA	Hyaluronic Acid
ECM	Extracellular Matrix
CNS	Central Nervous System
HMW	High Molecular Weight
RHAMM	Receptor for Hyaluronan Mediated Motility
HMMR	Hyaluronan Mediated Motility Receptor
LYVE1	Lymphatic Vessel Endothelial Hyaluronan Receptor 1
TLR	Toll-like Receptor
PNN	Perineuronal Net
IαI	Inter-alpha-inhibitor
HC	Heavy Chain
TSG-6	Tumor Necrosis Factor Stimulated Gene 6
NSC	Neural Stem Cell
PNS	Peripheral Nervous System
HAS	Hyaluronic Acid Synthase
SGZ	Subgranular Zone
SVZ	Subventricular Zone
MS	Multiple Sclerosis
CSPG	Chondroitin Sulfate Proteoglycan
CEMIP	Cell Migration Inducing and Hyaluronan Binding Protein
EAE	Experimental Autoimmune Encephalomyelitis
